# Thiosemicarbazone scaffold for the design of antifungal and antiaflatoxigenic agents: evaluation of ligands and related copper complexes

**DOI:** 10.1038/s41598-017-11716-w

**Published:** 2017-09-11

**Authors:** Dominga Rogolino, Anna Gatti, Mauro Carcelli, Giorgio Pelosi, Franco Bisceglie, Francesco Maria Restivo, Francesca Degola, Annamaria Buschini, Serena Montalbano, Donatella Feretti, Claudia Zani

**Affiliations:** 1 Department of Chemistry, Life Sciences and Environmental Sustainability and CIRCMSB (Consorzio Interuniversitario di Ricerca in Chimica dei Metalli nei Sistemi Biologici), Università di Parma, Parco Area delle Scienze, 43124 Parma, Italy; 2 Department of Medical and Surgical Specialities, Radiological Sciences and Public Health, University of Brescia, Viale Europa 11, 25123 Brescia, Italy

## Abstract

The issue of food contamination by aflatoxins presently constitutes a social emergency, since they represent a severe risk for human and animal health. On the other hand, the use of pesticides has to be contained, since this generates long term residues in food and in the environment. Here we present the synthesis of a series of chelating ligands based on the thiosemicarbazone scaffold, to be evaluated for their antifungal and antiaflatoxigenic effects. Starting from molecules of natural origin of known antifungal properties, we introduced the thio- group and then the corresponding copper complexes were synthesised. Some molecules highlighted aflatoxin inhibition in the range 67–92% at 100 μM. The most active compounds were evaluated for their cytotoxic effects on human cells. While all the copper complexes showed high cytotoxicity in the micromolar range, one of the ligand has no effect on cell proliferation. This hit was chosen for further analysis of mutagenicity and genotoxicity on bacteria, plants and human cells. Analysis of the data underlined the importance of the safety profile evaluation for hit compounds to be developed as crop-protective agents and at the same time that the thiosemicarbazone scaffold represents a good starting point for the development of aflatoxigenic inhibitors.

## Introduction

Food security and preservation is an ongoing major concern: it is in fact estimated that about 40% of the food produced worldwide is lost or spoiled. This not only reduces its availability, but, by forcing agricultural productivity, also has an impact on global climate change^1^. One of the most important cause of food spoilage is related to the presence of fungi, in particular of *Aspergillus*, *Penicillium*, *Fusarium* and *Alternaria* genera^2^. These fungi, in fact, are the principal producers of mycotoxins, and aflatoxins (AF) in particular, secondary metabolites with a severe toxic and carcinogenic potential. AF can lead to the induction of teratogenic, carcinogenic, oestrogenic, neurotoxic and immunosuppressive effects in humans and animals. They persist also in processed products, like milk or cheese, and represent therefore a great risk for human health^3^. AF can contaminate a wide variety of important agricultural products, causing important economic losses, and strict values are imposed for food consumption^4^.

The direct control of mycotoxin-producing fungi by using synthetic fungicides is still the most effective way to intervene, but it is well known that the extensive use of fungicides generates long term residues in food and in the environment^5^. Concerns on food safety and environmental health, combined with the global issue of emerging resistant pest strains, make urgent to develop novel crop-protective agents^6^. In this scenario, the exploitation of bioactive natural sources to obtain new agents with novel modes of actions may represent an innovative, successful strategy to minimize at the same time mycotoxin production and the use of harmful pesticides. Many natural products and their chemical analogues have been proposed as crop-protective agents^7^. Phenolic compounds with antioxidant activity, including eugenol, ferulic acid, vanillin and vanillylacetone, have been reported as AF inhibitors^8^. Moreover, recent studies have demonstrated the antifungal activities of some naturally occurring acetophenone derivatives^9^. On the other hand, inorganic substances, like copper salts, have been long used for their capacity of inhibiting the development of moulds and bacteria and can have effect on growth of *A*. *parasiticus* and aflatoxin production^10^. Some studies suggested that metal ions can influence the growth and the mycotoxin production of the toxigenic fungi *A*. *flavus* and *F*. *graminearum* and that this effect can be related to the ability of metal ions to intervene on the pattern of gene expressions of *A*. *flavus* ^9, 11^. The lipidic membrane that surrounds the cell constitutes a barrier to metal ions diffusion, but small hydrophobic molecules can easily diffuse through this barrier. Metal chelation could improve lipophilicity, facilitating the penetration of the complexes into lipid membranes, and, in this way, metal complexes should restrict proliferation of the microorganisms. Thiosemicarbazones represent a very attractive class of metal-chelating ligands for their coordinating versatility and the possibility to easily modify the molecular backbone and tuning their physical and chemical properties. They have a great variety of biological properties both as free ligands and as metal complexes^12^. Recently, we have disclosed the potential of some thiosemicarbazones for crop protection and food spoilage control, with a particular focus on the activity of these compounds against the two major genera of cereal mycotoxigenic fungi, i.e. *Fusarium* and *Aspergillus* ^13, 14^. Here we present the evaluation of other thiosemicarbazone ligands (**L1–L6**) for their antifungal and anti-aflatoxin activity towards *A*. *flavus*. Starting from molecules of natural origin, like vanillin and its derivatives, we introduced the thio- group in the perspective to obtain more potent compounds; copper complexes were then synthesised, with the aim to synergistically improve the capability of the free ligands to inhibit toxin production. The effects of **L1–L6** and of their copper complexes on fungal growth and aflatoxin biosynthesis were determined. With a view to use these compounds in field, an assessment of the cyto- and geno-toxic effects on healthy human cells, particularly on human cell lines deriving from the districts that can be exposed to chemicals (gastrointestinal tract, pulmonary epithelium and epidermis) was performed on the most active compounds. Finally, best hits were evaluated for their toxic and genotoxic activities on bacteria and plants cells.

## Materials and Methods

Chemicals were purchased from Sigma-Aldrich Srl (Milano, Italy). Dulbecco’s Modified Eagle’s medium (DMEM) and RPMI-1640 medium were purchased from Lonza Group Ltd (Basel, Switzerland); Ham’s Nutrient Mixture F-12 and Fetal bovine serum (FBS) were purchased from EuroClone S.p.a. (Milano, Italy). Hs27 (ATCC, CRL1634), CRL 1790 (ATCC, CCD 841 CoN) and HFL1(ATCC, CCL-153) were obtained from the American Type Culture Collection (ATCC). U937 cells were obtained from the American Tissue Culture Collection (Rockville, MD). CellTiter96® AQ_ueous_One Solution Cell Proliferation Assay was purchased from Promega Corporation, Madison, WI, USA.

### Chemistry

The purity of the compounds was determined by elemental analysis and verified to be ≥95%. ^1^H-NMR spectra were obtained in a 5 mm NMR precision tube at 298 K on a Bruker Avance 400 FT spectrophotometer. The ATR-IR spectra were recorded by means of a Nicolet-Nexus (Thermo Fisher) spectrophotometer by using a diamond crystal plate in the range of 4000–400 cm^−1^. Elemental analyses were performed by using a FlashEA 1112 series CHNS/O analyzer (Thermo Fisher) with gas-chromatographic separation. Electrospray mass spectral analyses (ESI-MS) were performed with an electrospray ionization (ESI) time-of-flight Micromass 4LCZ spectrometer. Samples were prepared in methanol. The MS spectra were recorded in methanol and acquired in positive EI mode by means of a DEP-probe (Direct Exposure Probe) mounting on the tip of a Re-filament with a DSQII Thermo Fisher apparatus, equipped with a single quadrupole analyzer. ICP data were obtained by mean of an emission spectrometer JY 2501 with coupled plasma induction in radial configuration HORIBA Jobin Yvon (Kyoto, Japan), ULTIMA2 model. Instrumental features: monochromator Model JY 2501; focal length 1 m; resolution 5 pm; nitrogen flow 2 l/min.

ICP source: nebulizer Meinhard, cyclonic spraying chamber; argon flow 12 l/min; wavelengths range 160–785 nm; optical bench temperature 32 °C. The wavelength used for quantitative analysis was chosen by examining the emission line with greater relative intensity, ensuring that there were no spectral interference with the Argon emission lines. Acquisition parameters: wavelength Cu (nm): 224.700; Voltage (V): 580; gain: 100. The quantitative analysis was performed after the acquisition of a calibration line using standard solutions in HNO_3_ at 2%, to simulate the final acidity of the samples; the concentration range of the standards varied from 1 mg/L to 100 mg/L. Compounds were dissolved in 10 mL of CH_3_OH (2% HNO_3_). Data acquisition and processing were performed using the ICP JY v 5.2 software (Jobin Yvon).

**Figure 1:**
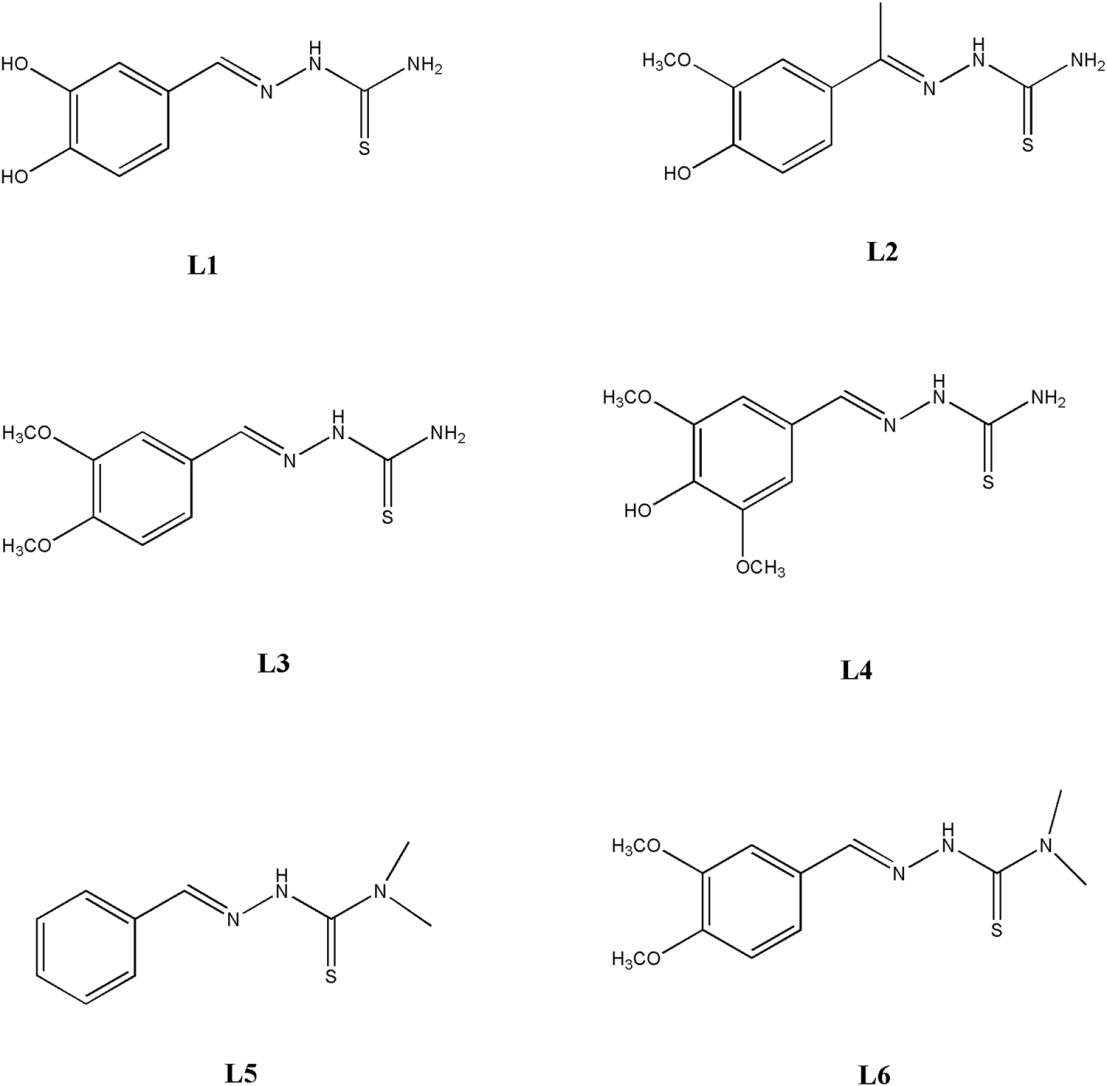
Thiosemicarbazone ligands **L1–L6**.

The synthesis of **L1–L6** (Fig. 1) was performed by using a procedure previously reported^15^. The aldehyde was dissolved in a hot ethanol solution containing few drops of glacial acetic acid. An equimolar amount of the appropriate thiosemicarbazide was added to the solution and the reaction was heated under reflux for 24 h. The solution was cooled r.t. and the ligands were obtained as precipitates. After filtration the solid was washed several times with cold ethanol and ether and then dried under vacuum.

#### N’-(3,4-dihydroxybenzyliden)thiosemicarbazone **L1**

Brown solid. Yield: 81%. ^1^H-NMR (DMSO-d_6_, 25 °C), δ: 11.20 (s, 1 H, NNH); 9.47, 9.01 (2 s, 1 H + 1 H, OH); 8.04, 7.71 (2 s, 1 H + 1 H, NH_2_); 7.89 (s, 1 H, CH = N); 7.17 (s, 1 H, CH_Ar_); 7.00 (d, 1 H, J = 8 Hz, CH_Ar_); 6.74 (d, 1 H, J = 8 Hz, CH_Ar_). EI-MS: m/z = 211.0 [M + H]^+^.

#### N’-(3-metoxy-4-hydroxy-acetophenone)thiosemicarbazone **L2**

Yellow solid. Yield: 87%. ^1^H-NMR (DMSO-d_6_, 25 °C), δ: 10.03 (s, 1 H, NNH); 9.34 (s, 1 H, OH); 8.22, 7.87 (2 s, 1 H + 1 H, NH_2_); 7.49 (d, 1 H, J = 2 Hz, CH_Ar_); 7.26 (dd, 1 H, J = 8 Hz, J’ = 2 Hz, CH_Ar_); 6.76 (d, 1 H, J = 8 Hz, CH_Ar_); 3.84 (s, 3 H, OCH_3_); 2.25 (s, 3 H, CH_3_). EI-MS: m/z = 240.0 [M + H]^+^.

#### N’-(3,4-dimetoxybenzyliden)thiosemicarbazone **L3**

Pink solid. Yield: 64%. ^1^H-NMR (DMSO-d_6_, 25 °C), δ: 11.18 (s, 1 H, NNH); 9.48, 8.99 (2 s, 1 H + 1 H, OH); 8.30 (t_broad_, 1 H, NH_Et_); 7.89 (s, 1 H, CH = N); 7.21 (d, 1 H, J = 2 Hz, CH_Ar_); 6.99 (dd, 1 H, J = 8 Hz, J’ = 2 Hz, CH_Ar_); 6.75 (d, 1 H, J = 8 Hz, CH_Ar_); 3.55 (m, 2 H, J = 7 Hz, CH_2_); 1.12 (t, 3 H, J = 7 Hz, CH_3_). EI-MS: m/z = 240.0 [M + H]^+^.

#### N’-(3,5-dimetoxy-4-hydroxybenzyliden)thiosemicarbazone **L4**

Brown solid. Yield: 90%. ^1^H-NMR (DMSO-d_6_, 25 °C), δ: 11.32 (s, 1 H, NNH); 8.80 (s, 1 H, OH); 8.13, 7.99 (s + s, 1 H + 1 H, NH_2_); 7.92 (s, 1 H, CH = N); 7.05 (s, 1 H, CH_Ar_); 3.81 (s, 6 H, OCH_3_). EI-MS: m/z = 255.0 [M + H]^+^.

#### N’-(benzyliden)-4,4-dimethylthiosemicarbazone **L5**

Yellow solid. Yield: 57%. ^1^H-NMR (DMSO-d_6_, 25 °C), δ: 10.95 (s, 1 H, NNH); 8.20 (s, 1 H, CH = N); 7.64 (d, 2 H, J = 7 Hz, CH_Ar_); 7.38 (m, 3 H, CH_Ar_); 3.30 (s, 6 H, NCH_3_). EI-MS (C_10_H_13_N_3_S, CH_3_OH): m/z = 207.0 [M + H]^+^.

#### N’-(3,4-dimetoxybenzyliden)-4,4-dimethylthiosemicarbazone **L6**

Red solid. Yield: 61%. ^1^H-NMR (DMSO-d_6_, 25 °C), δ: 10.84 (s, 1 H, NNH); 8.10 (s, 1 H, CH = N); 7.25 (s, 1 H, CH_Ar_); 7.12 (d, 1 H, J = 8.4 Hz, CH_Ar_); 6.99 (d, 1 H, J = 8.4 Hz, CH_Ar_); 3.79 (s, 6 H, NCH_3_). EI-MS (C_12_H_17_N_3_SO_2_, CH_3_OH): m/z = 268.0 [M + H]^+^.

#### General method for the synthesis of copper complexes ***1***–***6***

100 mg (2 eq.) of the thiosemicarbazone ligand were dissolved in 10 ml of degassed methanol. 1 eq. of CuCl_2_·2H_2_O was dissolved in 5 mL of degassed methanol and this solution was added to the previous one. The mixture was stirred at room temperature for 4 hours under N_2_. Then, it was cooled overnight; the precipitate was filtered off and washed with ether.

#### Cu_3_(**L1**)(**L1**-H)Cl_2_ (**1**)

Orange powder. Yield = 25%. ^1^H-NMR (DMSO-d_6_, 25 °C), δ: 11.68 (s, 1 H, NNH); 9.62, 9.04 (2 s, 1 H + 1 H, OH); 8.57, 8.37 (2 s, 1 H + 1 H, NH_2_); 7.97 (s, 1 H, CH = N); 7.24 (s, 1 H, CH_Ar_); 7.07 (d, 1 H, J = 9 Hz, CH_Ar_); 6.74 (d, 1 H, J = 8.5 Hz, CH_Ar_). ESI-MS (CH_3_OH): m/z = 485 (100, [ML_2_]^+^), (50, 332 [MLCl + Na]^+^). Anal. calcd. for C_16_H_17_N_6_S_2_O_4_Cu_3_Cl_2_: C 28.14, H 2.51, N 12.30. Found: C 28.19, H 2.37, N 12.32. ICP: Cu found 28.9%, calcd. 27.9%.

#### Cu_2_(**L2**)Cl_2·_H_2_O (**2**)

Green powder. Yield = 28%. ^1^H-NMR (DMSO-d_6_, 25 °C) δ: 10.48 (s, 1 H, NNH); 9.43 (s, 1 H, OH); 8.79, 8.42 (2 s, 1 H + 1 H, NH_2_); 7.55 (s, 1 H, CH_Ar_); 7.33 (d, 1 H, J = 8.5 Hz, CH_Ar_); 6.78 (d, 1 H, J = 8.5 Hz, CH_Ar_); 3.85 (s, 3 H, OCH_3_); 2.37 (s, 3 H, CH_3_). ESI-MS (CH_3_OH): m/z = 541 (100, [ML_2_]^+^), 302 (40, [ML]^+^). Anal. calcd. for C_10_H_13_N_3_SO_2_Cu_2_Cl_2_ + H_2_O: C 26.39, H 3.32, N 9.23. Found: C 25.81, H 2.87, N 8.93. ICP: Cu found 29.5%, calcd. 27.9%.

#### Cu_3_(**L3**)(**L3**-H)Cl_2_ (**3**)

Yellow powder. Yield = 28%. ^1^H-NMR (DMSO-d_6_, 25 °C) δ: 11.76 (s, 1 H, NNH); 8.65, 8.58 (2 s, 1 H + 1 H, NH_2_); 7.55 (s, 1 H, CH_Ar_); 8.05 (s, 1 H, CH = N); 7.58 (s, 1 H, CH_Ar_); 7.21(d, 1 H, J = 8.5 Hz, CH_Ar_); 6.98 (d, 1 H, J = 8.5 Hz, CH_Ar_); 3.83, 3.80 (2 s, 3 H + 3 H, OCH_3_). ESI-MS (CH_3_OH): m/z = 541 (90, [ML_2_]^+^), 302 (100, [ML]^+^). Anal. calcd. for C_20_H_25_N_6_S_2_O_4_Cu_3_Cl_2_: C 32.51, H 3.41, N 11.37. Found: C 32.71, H 3.55, N 11.44. ICP: Cu found 26.1%, calcd. 25.8%.

#### Cu_3_(**L4**)(**L4**-H)Cl_2_ 2H_2_O(**4**)

Yellow powder. Yield = 37%. ^1^H-NMR (DMSO-d_6_, 25 °C) δ: 11.81 (s, 1 H, NNH); 8.95 (s, 1 H, OH); 8.70, 8.66 (2 s, 1 H + 1 H, NH_2_); 8.01 (s, 1 H, CH = N); 7.13 (s, 1 H, CH_Ar_). ESI-MS (CH_3_OH): m/z = 573 (100, [ML_2_]^+^). Anal. calcd. for C_20_H_25_N_6_S_2_O_6_Cu_3_Cl_2 + _2H_2_O: C 29.77, H 3.62, N 10.41. Found: C 29.45, H 3.49, N 10.47. ICP: Cu found 21.8%, calcd. 23.6%.

#### Cu(**L5**′)Cl (**5**)

Yellow powder. Yield = 33%. ^1^H-NMR (DMSO-d_6_, 25 °C) δ: 7.78 (m, 2 H, CH_Ar_); 7.48 (m, 3 H, CH_Ar_) 3.84, 3.81 (2 s, 3 H + 3 H, N(CH_3_)_2_). ESI-MS (CH_3_OH): m/z = 476 (40, [ML_2_ + H]^+^), 270 (100, [ML + H]^+^).

Crystals of **L5**′, a cyclized form of **L5**, suitable for X-ray diffraction analysis were obtained during some attempts to recrystallize **5**; crystals of **5**′ suitable for X-ray diffraction analysis were obtained by vapour diffusion of ether in a saturated DMF solution of **5**.

#### Cu(**L6**′)Cl (**6**)

Yellow powder. Yield = 37%. ^1^H-NMR (DMSO-d_6_, 25 °C) δ: 7.29 (d, 1 H, J = 8 Hz, CH_Ar_); 7.06 (d, 1 H, J = 8 Hz, CH_Ar_); 7.00 (s, 1 H, CH_Ar_); 4.16, 4.09 (2 s, 3 H + 3 H, OCH_3_); 3.83, 3.80 (2 s, 3 H + 3 H, N(CH_3_)_2_). ESI-MS (CH_3_OH): m/z = 596 (50, [ML_2_]^+^), 330 (100, [ML]^+^). ICP: Cu found 18.7%, calcd. for C_12_H_15_N_3_O_2_SCuCl: 19.3%. Crystals of **6**′ suitable for X-ray diffraction analysis were obtained by vapour diffusion of ether in a saturated DMF solution of **6**.

### X-ray structures

**L5**′ and **6**′ were collected at 100 K under nitrogen flux at Elettra Sincrotrone (Trieste, Italy) on beamline XRD1 with a wavelength of 0.7 Å (NdBFe Multipole Wiggler, Hybrid linear, 4.27 keV with a power of 8.6 kW, source size full width half maximum (fwhm) beam size at sample of 2.0 × 0.37 mm, 0.7 × 0.2 mm, and photon flux 1012–1013 ph/s), Dectris Pilatus 2 M detector. Data were reduced with CrysalisPro software^16^. For **5**′, single crystal X-ray diffraction analysis was performed on a SMART APEX2 diffractometer using Mo Kα radiation (λ = 0.71073 Å, Lorentz polarization and absorption correction applied) at room temperature (293 K). The SAINT^17^ software was used for integration of reflection intensity and scaling, SADABS^18^ for absorption correction. A semi-empirical absorption correction, based on multiple scanned equivalent reflections, has been carried out and gave 0.3658 < T < 0.7459). Structures were solved by direct methods using SIR97^19^ and refined by full-matrix least-squares on all F2 using SHELXL97^20^ implemented in the WinGX package^21^. For all the structures, anisotropic displacement parameters were refined except for hydrogen atoms. Hydrogen atoms were introduced in calculated positions riding on their carrier atoms.

**L5**′. The crystal system is orthorhombic, space group *Pna*2_1_, cell parameters *a* = 15.4877(2), *b* = 5.8132(1), *c* = 11.1034(1) Å, *V* = 999.67(2) Å^3^. The asymmetric unit is formed by a single molecule of formula C_10_H_12_Cl_2_N_3_S, *M* = 206.29 Da, *Z* = 4, *D*_*c*_ *= *1.37 g cm^−3^, *μ* = 2.85 mm^−1^, *F*(000) = 436. A total of 17969 reflections were collected up to a *θ* range of 33.10° (±22 *h*, ±8 *k*, ±15 *l*), 3388 unique reflections (*R*_*int*_ = 0.038).

**5**′. The crystal system is triclinic, space group *P-1*, cell parameters *a* = 8.124(3), *b* = 8.477(3), *c* = 9.761(4) Å, α = 100.121(6), β = 114.531(5), γ = 98.713(6)°, *V* = 582.8(4) Å^3^. The asymmetric unit is formed by half a molecule of formula C_10_H_11_ClCu_0.5_N_3_S, *M* = 272.50 Da, *Z* = 2, *D*_*c*_ *= *1.55 g cm^−3^, *μ* = 13.66 mm^−1^, *F*(000) = 279. A total of 7598 reflections were collected up to a *θ* range of 27.39° (±10 *h*, ±10 *k*, ±12 *l*), 2632 unique reflections (*R*_*int*_ = 0.044).

**6**′. The crystal system is monoclinic, space group *P*2_1_/*n*, cell parameters *a* = 9.0147(2), *b* = 13.5881(3), *c* = 11.5443(2) Å, β = 105.418(2)°, *V* = 582.8(4) Å^3^. The asymmetric unit is formed by half a molecule of formula C_12_H_15_ClCu_0.5_N_3_O_2_S, *M* = 332.55 Da, *Z* = 4, *D*_*c*_ *= *1.62 g cm^−3^, *μ* = 11.92 mm^−1^, *F*(000) = 686. A total of 12038 reflections were collected up to a *θ* range of 29.99° (±12 *h*, ±17 *k*, ±16 *l*), 2632 unique reflections (*R*_*int*_ = 0.043).

All the non-hydrogen atoms in the molecules were refined anisotropically. The hydrogen atoms were partly found and partly placed in the ideal positions using riding models.

CCDC 1556287, 1556288 and 1556289 contain the supplementary crystallographic data (see also the attached CIF file). These data can be obtained free of charge from The Cambridge Crystallographic Data Centre via http://www.ccdc.cam.ac.uk/data_request/cif.

### *A. flavus* strains

A toxigenic and an atoxigenic strain of *A*. *flavus* were used^14^.

### Effect on *A*. *flavus* growth

Conidia of *A*. *flavus* strains obtained from 10-day YES-agar [2% (w⁄v) yeast extract (Difco, Detroit, MI), 5% (w⁄v) sucrose (Sigma, St Louis, MO), 2% (w⁄v) agar (Difco)] cultures were quantified by OD_600_, and viability (>90%) was determined according to previously disclosed methods^22^. Conidial germination rate and post-germination hyphal outgrowth were assessed by analyzing changes in optical density of spore suspensions after 38–46 h: in a 96 well microtiter plate (Sarstedt, Newton, NC, USA) 5 × 10^3^ spores were inoculated in a final volume of 200 μL of YES liquid medium amended with molecules (50 or 100 μM), and incubated statically at 28 °C. DMSO (0.5% and 1% respectively) was used as control. The optical density at 620 nm was recorded for each well with a microplate reader (MULTISKAN EX, Thermo Electron Corporation, Vantaa, Finland) without shaking. Samples were inoculated in quadruplicate.

### Effect on aflatoxin accumulation

The high throughput procedure described in our previous works^22, 23^ was used to assess aflatoxin accumulation in a coconut-milk derived medium (CCM). Briefly, suspensions of conidia were diluted and brought to the final concentration of 5 × 10^2^ conidia/well; cultures were set in a final volume of 200 μL/well of CCM medium added with molecules at 50 or 100 μM. DMSO (0.5% and 1% respectively) was used as control. The plates were incubated in the dark under stationary conditions for 6 days at 25 °C. Aflatoxin accumulation was monitored by fluorescence emission determination: readings were performed directly from the bottom of wells of the culture plate with a microplate reader (TECAN SpectraFluor Plus, Männedorf, Switzerland) using the following parameters: λ_ex_ = 360 nm; λ_em_ = 465 nm; manual gain = 83; lag time = 0 μs; number of flashes = 3; and integration time = 200 μs. Samples were inoculated in quadruplicate.

### Statistical analysis

For statistical analyses one-way analysis of variance (ANOVA) was used in the Past 3.x software. Results of mycelial growth and aflatoxin accumulation were analyzed by Tukey’s test; differences were considered significant at p < 0.001.

### Cytotoxicity

The antiproliferative effect of the compounds was evaluated by MTS assay (CellTiter96® AQ_ueous_ One Solution Cell Proliferation Assay) towards different human cell lines: Hs27 foreskin fibroblasts, CRL1790 colon epithelial, HFL1 lung fibroblasts and U937 histiocytic lymphoma cells. Hs27 and CRL1790 were cultured in DMEM supplemented with 10% (v/v) fetal bovine serum, 1% L-glutamine (2 mM) and 1% penicillin (100 units mL^−1^)/streptomycin (100 μg mL^−1^). HFL1were cultured in Ham’s Nutrient Mixture F-12 with L-Glutamine supplemented with 10% (v/v) fetal bovine serum and 1% penicillin (100 units mL^−1^)/streptomycin (100 μg mL^−1^). U937 cells were cultured in RPMI-1640 medium supplemented with 10% (v/v) fetal bovine serum, 1% L-glutamine (2 mM) and 1% penicillin (100 units mL^−1^)/streptomycin (100 μg mL^−1^). Hs27, CRL1790 and HFL1cells were used between passage numbers 5 and 20. Cells were maintained in a humidified atmosphere at 5% CO_2_ and 37 °C and culture medium was refreshed every two or three days during sub-culturing.

The cytotoxicity was evaluated according to the following method: 5 × 10^3^ cells/well were seeded in 96-well plates in 100 μL of medium without phenol red with 5% fetal bovine serum and then incubated at 37 °C in a humidified (95%) CO_2_ (5%) incubator. After 24 h, cells were treated, in quadruplicate, with increasing concentrations of the molecules in the range 0.5 to 100 µM for further 24 h. The assay was performed by adding 20 μL of the CellTiter96® AQ_ueous_ One Solution Cell Proliferation Assay directly to the culture wells, incubating for 4 h and then recording the absorbance at 485 nm with a 96-well plate reader (TECAN SpectraFluor Plus, Männedorf, Switzerland). MTS assay was performed to identify GI_50_ value, that is the concentration of drug that causes a 50% reduction of cell growth^24^.

### Genotoxicity on human cells

To assess primary DNA damage the alkaline version of Comet assay was performed with U937 cells as described in a previously published work^25^. Briefly, the cells were seeded at a concentration of 1 × 10^5^ cell/mL in 24-well plates in 1 mL of medium, supplemented with 1% glutamine, 1% penicillin/streptomycin and 10% fetal bovine serum and then incubated at 37 °C in a humidified (95%) CO_2_ (5%) incubator. After 24 h cells were treated, in duplicate, with increasing concentrations of the molecules in the range 25 to 100 µM for 1 h. After treatment, determinations of cell numbers and viabilities were performed with the trypan blue exclusion method. Only the treatments that had a viability higher than 70% have been processed in the Comet assay. Positive and negative controls were represented by ethylmethanesulfonate (EMS), 2 mM, and DMSO, 100 µM, respectively. DNA was stained with 75 μL ethidium bromide (10 μg/mL) before the examination at 400 × magnification under a Leica DMLS fluorescence microscope (excitation filter BP 515–560 nm, barrier filter LP 580 nm), using an automatic image analysis system (Comet Assay IV – Perceptive Instruments Ltd, UK). The “IBM SPSS Statistics 24” software was used to analyze statistical differences between samples. The mean values from the repeated experiments were used in a one-way analysis of variance (ANOVA). If significant F-values (P < 0.05) were obtained, Student’s t test (Bonferroni’s version) was performed.

### Mutagenicity assessment of L5 on bacteria and plants cells

The samples in DMSO underwent the *Salmonella*/microsome test (Ames test) at increasing doses, with *S. typhimurium* TA98 and TA100 strains, with and without metabolic activation (S9 mix) to highlight the presence of indirect and direct mutagenic substances. A range of doses from 0.1 to 100 µM/plate was applied. TA98 strain detects frame-shift mutagens and TA100 strain responds to base-pair substitution mutations^26^. For positive control, 2-nitrofluorene for TA98 without S9 (10 μg/plate), sodium azide for TA100 without S9 (10 μg/plate), and 2-aminofluorene for both strains with S9 mix (20 μg/plate) were used, respectively. DMSO was used as negative control. The results were expressed as number of revertants/plate. All experiments were conducted in duplicate. The data obtained were the average of duplicate plates and were expressed as mutagenicity ratio, dividing the revertants/plate by the spontaneous mutation rate. Results were considered positive if two consecutive dose levels or the highest non-toxic dose level produced a response at least twice that of the solvent control, and at least two of these consecutive doses showed a dose-response relationship^27, 28^.

*A. cepa test:* in a preliminary toxicity assay, equal-sized young bulbs of onion were exposed for 72 hours in the dark to different concentrations (from 0.1 to 100 µM) of each molecules. Root length was used to calculate the EC_50_ value of the compound^29, 30^ and to identify the concentrations to be used in the *A. cepa* genotoxicity assay. Other macroscopic parameters (turgescence, consistency, change in colour, root tip shape) were used as toxicity indexes^29, 30^.

The *A. cepa* micronucleus test was performed using equal-sized young bulbs per sample^31^. After 72-hour pre-germination in Rank solution, the bulbs were exposed to 4 doses of compounds (10–100 µM) for 24 hours. Negative (Rank solution + DMSO, 24 h) and positive (maleic hydrazide 10 mg/l, 6 h) controls were performed. After exposure, the roots remained in recovery time for 44 hours and were then fixed in Carnoy’s solution. For microscopic analysis 5000 cells/sample were scored for mitotic index (as a measure of cellular division and therefore of sample toxicity) and 10000 cells/sample were scored for micronucleus frequency. The results were reported as number of micronuclei per 100 cells and the data were analysed by using χ^2^ and Dunnett’s tests. *A. cepa* aberration test was carried out according to Cabaradvic^32^, based on Fiskesjo method with minor modifications^29, 30^. After 72-hour pre-germination in Rank solution, the bulbs were exposed to 4 doses of compounds (10–100 µM) for 24 h. After treatment, roots were fixed in Carnoy’s solution. 5 slides for sample were prepared by using Feulgen technique for analyse mitotic index (MI), mitosis distribution and type and frequency of chromosomal aberrations (structural and numerical ones) in different mitosis phases. For mitotic index evaluation 5000 cells for samples were scored; for chromosomal aberration 1000 cells in division cycle for sample (metaphase, anaphase and telophase) were scored. Statistical analysis was performed using analysis of variance (ANOVA) for mitotic index and Mann-Whitney test for chromosomal aberration. All *A. cepa* experiments were performed in duplicate (two independent assays).

## Results

### Chemistry

**Figure 2:**
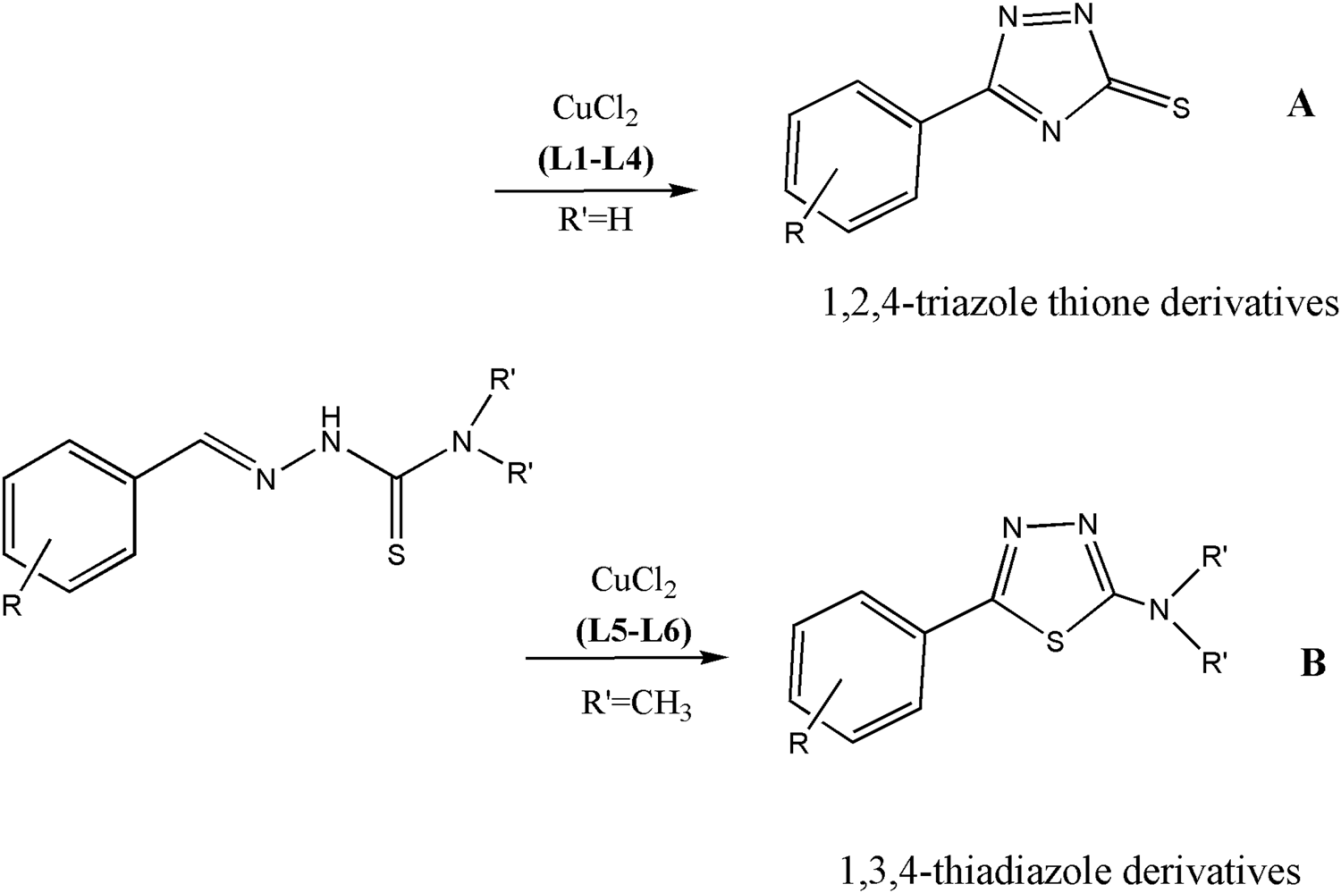
Possible cyclisation products for thiosemicarbazones **L1–L6**.

Ligands **L1–L6** (Fig. 1) were synthesised by condensation between an aldehyde and thiosemicarbazide or 4,4-dimethyl-3-thiosemicarbazide^15^. All ligands were characterised by the usual spectroscopic techniques and data are reported in the Experimental Section. Although for these ligands it is possible *E/Z* isomerisation around the C = N double bond, the ^1^H-NMR spectra of **L1–L6** registered in d_6_-DMSO showed just one set of signals, that can be related to the *E* isomer. **L1–L4** are reacted with CuCl_2_ leading to the isolation of the copper(I) complexes **1**–**4**: ^1^H-NMR, IR, mass, ICP and elemental analysis data are reported in the Experimental Section. All the isolated copper compounds are stable at room temperature, non-hygroscopic and insoluble in water, as well as in the common organic solvents, but readily soluble in DMF and DMSO. The ^1^H-NMR spectra of **1–4** present sharp signal, relative to diamagnetic Cu(I) complexes (Fig. S1). In the protonic spectra of **1–4**, both the signal relative to the NH, the NH_2_ and the iminic proton are shifted to lower fields, as can be seen in Figure S1. In order to better clarify the redox process involving the metal centre, the mother liqueur, obtained after filtration of complex **3**, was completely evaporated and the ^1^H-NMR of the corresponding crude product is reported in Figure S2. In this case, the protonic spectrum lacks of the signals relative to both the iminic and the NH_2_ proton and data are in accord with the presence of a cyclized form of the ligand, as a result of an intramolecular oxidative cyclisation of the thiosemicarbazone ligand (Fig. 2), as discussed in the following paragraph. In the IR spectra of **1**–**4** there is a strong band between 3500 and 3000 cm^−1^, relative to the symmetric and asymmetric stretching mode of NH_2_. ESI-mass spectra of **1–4** are reported in Figures S3–S6.

A different behaviour is observed when **L5** and **L6** are reacted with copper(II). Again, ^1^H-NMR spectra of the corresponding complexes **5** and **6** reveal sharp signal, indicating the presence of diamagnetic Cu(I) coordination compounds. The absence of signals relative to the iminic and the NH protons in the 8–10 ppm range is in accord with the presence of a cyclized form of the ligand coordinated to the metal centre.

During attempts to crystallize **5**, some crystals of the cyclized form of **L5** (indicated as **L5**′) were serendipitously obtained and characterized by X-ray diffraction analyses (see below). The experimental data obtained for **5** and **6** support the proposed structure Cu(I)(cyclized-ligand)Cl.

**Figure 3:**
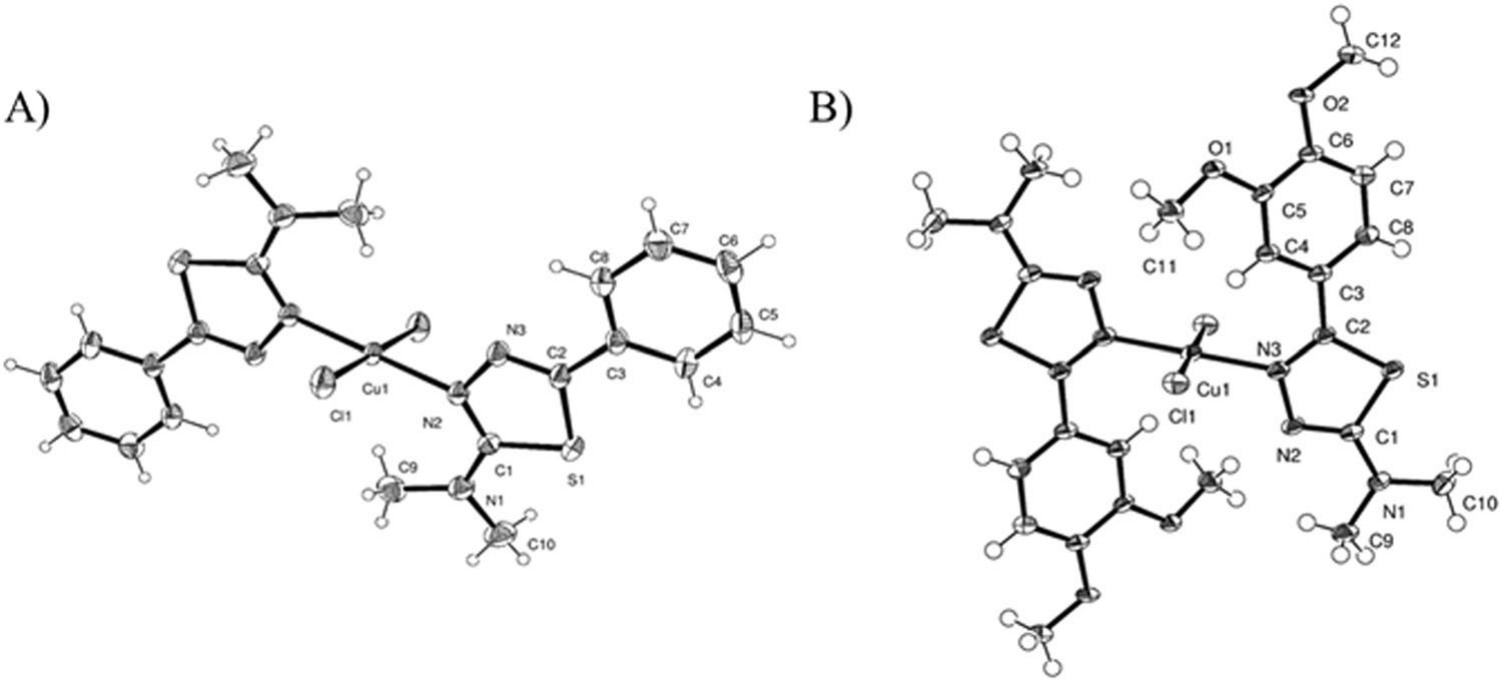
ORTEP representation of **5**′ (**A**) and **6**′ (**B**) with ellipsoids at 50% probability.

Slow vapour diffusion of ether in a saturated DMF solution of **5** and **6** led to the isolation of the Cu(II) complexes **5**′ and **6**′, whose crystal structures were determined by X-ray diffraction analysis on single crystal, as detailed in the discussion section (Fig. 3). Oxidation undergone by **5** and **6** occurred in solution during recrystallisation. This is confirmed also by X-ray powder diffraction analysis: the traces of **5** and **6**, in fact, do not match with the calculated spectra of **5**′ and **6**′, indicating that the powders are different chemical species respect to the ones subsequently obtained by re-crystallisation.

### Fungal growth and aflatoxin inhibition

Both ligands **L1–L6** and copper complexes **1**–**6** were tested at different concentrations (10, 25, 50 and 100 μM) for their ability to inhibit growth and AF accumulation in *A*. *flavus*. DMSO was used as control. In Table 1 data concerning the effects of 50 and 100 μM treatments on growth and AF inhibition are reported. **L1** and **L4** do not affect growth neither at 50 μM nor at 100 μM concentration. **L3** and **L6** were provided with a scarce fungistatic activity (less than 30% growth inhibition at the higher dose), but **L2** (at 50 μM) and **L5** (at 100 μM) halved the biomass increase.

**Table 1:** Antifungal and anti-aflatoxigenic activities for **L1–L6** and for copper complexes **1**–**6** at 50 and 100 μM concentration: results are expressed respectively as mean percentage inhibition of growth and of aflatoxin production (in comparison with non-treated controls; mean ± SD).

Compound	Growth Inhibition (%)		Aflatoxin Inhibition (%)	
	50 μm	100 μm	50 μm	100 μm
**L1**	0.6 ± 0.3	0.5 ± 0.6	6.1 ± 0.8	1.1 ± 0.3
**L2**	47.5 ± 2.5*	51.7 ± 2.3*	26.7 ± 2.4*	17.7 ± 9.5*
**L3**	25.9 ± 1.6*	25.6 ± 2.3*	24.8 ± 3.0*	25.7 ± 4.7*
**L4**	0.4 ± 0.4	0.7 ± 0.6	13.8 ± 1.8*	20.7 ± 1.4*
**L5**	23.8 ± 1.5*	43.9 ± 1.9*	78.0 ± 6.9 *	92.3 ± 4.2*
**L6**	5.8 ± 0.8*	21.8 ± 1.7*	41.8 ± 2.7*	48.6 ± 1.4*
**1**	—	—	0.6 ± 0.7	6.6 ± 0.4
**2**	59.7 ± 1.4*	73.6 ± 3.0*	52.0 ± 3.3*	72.7 ± 3.9*
**3**	40.5 ± 1.3*	51.0 ± 1.9*	61.1 ± 2.4*	79.0 ± 2.2*
**4**	28.0 ± 0.6*	62.7 ± 2.4*	48.8 ± 2.2*	62.1 ± 1.4*
**5**	0.6 ± 0.3	1.1 ± 0.4	36.6 ± 2.0*	67.3 ± 2.1*
**6**	1.0 ± 0.3	6.2 ± 0.9*	60.7 ± 1.2*	67.8 ± 3.4*

Statistical differences between treated and non-treated samples were reported. (*) p-value < 0.001.

As far as the effect of the ligands on AF biosynthesis, **L5** and **L6** only were provided with a relevant inhibitory activity. Mycotoxin biosynthesis was nearly halved when fungal cultures were treated with either at 50 or 100 μM doses of **L6**. AF accumulation in the medium was 90% reduced in the 100 μM **L5** amended cultures. The anti-aflatoxigenic efficacy of **L5** was considerable even if its concentration was lowered to 50 μM (76% inhibition). Moreover, **L5** displayed a moderate fungistatic activity (43 and 22% at 100 and 50 μM, respectively).

Generally, the copper complexes showed a better activity profile then uncomplexed ligands: the metal complexes **2**, **3**, **4** and **6** have aflatoxin inhibition ranges from 61 to 80% at 100 μM and from 35 to 62% at 50 μM. Exceptions are represented by **1** and also by **5**, for which a lower AF inhibition percentage respect to the starting ligand **L5** was observed (Table 1). The inhibition activity on aflatoxin production is accompanied for complexes **2–4** also by fungal growth inhibition (from 51 to 72% at 100 μM, Table 1).

### Cytotoxicity

Compounds **2**, **3**, **5**, **6** and **L5**, the most promising ones in term of activity, were tested for their cytotoxicity against a panel of human cell lines. Growth inhibition (GI) determination was performed by MTS assay; data are calculated as a mean of four independent experiments and are shown in Table 2. In Figure S10 the representative dose-response curves for **L5** and the copper complex **5** are shown. Unfortunately, all the copper complexes showed important cytotoxicity in the micromolar range, while **L5** has very no effect on cell proliferation. Therefore, only **L5** was chosen for further analysis of mutagenicity and genotoxicity on bacteria, plants and human cells.

**Table 2:** GI_50_ value, concentration of drug that causes a 50% reduction of cell growth, obtained for the most antimycotoxigenic compounds on different human cell lines.

Compound (µM)	Crl1790	Hs27	HFL1	U937
**L5**	>100	>100	>100	>100
**2**	31	16	30	27
**3**	38	17	29	27
**5**	3	3	1	4
**6**	1	3	1	3

### Genotoxicological assessment on bacteria, plants and human cells

Three different tests were carried out on **L5** in order to assess its ability to induce genetic damage in target cells of different organisms, i.e. bacteria, plant and human cells. The mutagenic activity (induction of gene mutation) was studied using Ames test with the *S*. *typhimurium* strains TA100 and TA98, with and without microsomal activation (S9 fraction). **L5** was tested in a dose range from 0.1 to 100 µM/plate and the results of the tests were expressed as Mutagenicity Ratio (MR), obtained from the mean number of revertants colonies per plates for negative controls: it is worth of note that **L5** exhibited no mutagenicity in the bacterial test on *S*. *typhimurium* TA100 and TA98 strains with or without metabolic activation at all tested doses (Table 3).

**Table 3:** Mutagenicity data in *S. typhimurium* TA98 and TA100 strains treated with **L5**, with and without S9 activation. Results are expressed as revertants/plate (mean ± standard deviation) and mutagenicity ratio (MR).

DOSE (µM/plate)	TA98-S9		TA98 + S9		TA100-S9		TA100 + S9	
	mean ± SD	MR	mean ± SD	MR	mean ± SD	MR	mean ± SD	MR
**Negative control**	19.0 ± 6.4		36.2 ± 6.0		109.5 ± 9.9		127.3 ± 8.1	
**0**.**1**	10.0 ± 2.8	**0**.**5**	34.0 ± 1.4	**0**.**9**	128.0 ± 14.1	**1**.**2**	143.0 ± 7.1	**1**.**1**
**1**	15.5 ± 7.8	**0**.**8**	33.5 ± 4.9	**0**.**9**	116.0 ± 11.3	**1**.**1**	132.0 ± 9.9	**1**.**0**
**10**	17.0 ± 2.8	**0**.**9**	35.0 ± 4.2	**1**.**0**	105.5 ± 6.4	**0**.**9**	132.5 ± 7.8	**1**.**0**
**50**	21.0 ± 2.8	**1**.**1**	36.5 ± 0.7	**1**.**0**	135.5 ± 20.5	**1**.**1**	94.5 ± 2.1	**0**.**7**
**100**	17.0 ± 4.2	**0**.**9**	40.0 ± 5.7	**1**.**1**	105.0 ± 1.4	**1**.**0**	138.0 ± 5.7	**1**.**1**

Positive controls for TA98 (±S9) and TA100 (±S9): >1000.

*A*. *cepa* test showed toxicity on roots at 100 µM, therefore micronuclei analysis at this dose cannot be performed; for lower doses no micronuclei increase was observed (Table 4). The chromosome aberration test in *A*. *cepa* was carried out on all doses (10, 25, 50 and 100 µM) and statistically significant increase of aberrations was observed only at the dose of 25 µM, but no dose response curve was found. The higher concentration did not induce genotoxic effects, but the highest dose (100 µM) showed a light toxic effect expressed by a lower mitotic index (8% vs 11.7% of negative control), which confirmed the results reported for micronuclei test.

**Table 4:** Micronuclei frequency (MCN), mitotic index (MI) and frequency and type of aberration in *A*. *cepa* roots treated with **L5**. *p < 0.05; **p < 0.01; ***p < 0.001.

L5 (µM)	MCN (mean ± SD)	Mitotic index (%)	Aberration frequencies in different cell cycle phases (%)			
			metaphase	anaphase	telophase	Total cells in division
10	0.6 ± 0.55	9.7	7.1	13.8	1.2	7.5
25	1.2 ± 1.1	9.8	8.3	25.9*	3.1	14.0***
50	0.8 ± 1.3	11.9	1.1	9.0	0.4	2.9
100	tox	8**	11.5*	20.5	0.7	7.9
**Negative control**	1.6 ± 1.5	11.7	4.6	12.3	1.4	5.7

**Figure 4:**
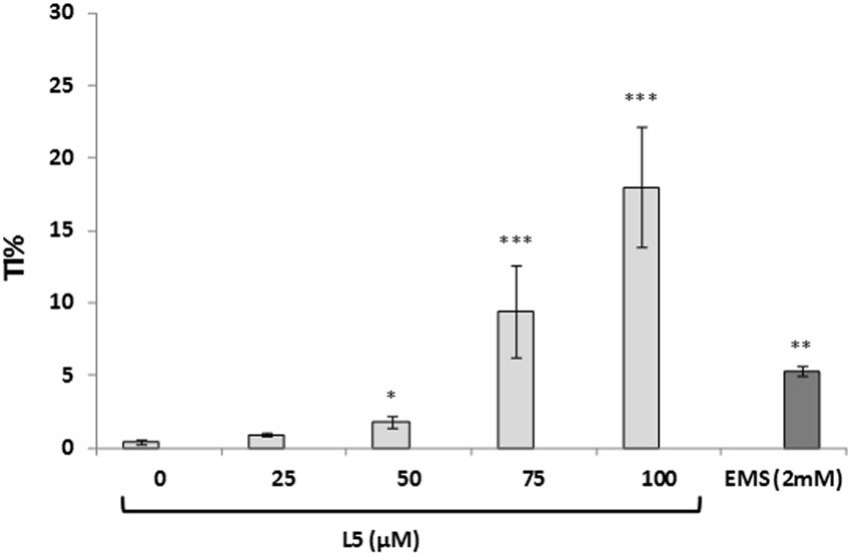
Comet assay: DNA damage induced by **L5** on U937 cells treated for 1 h. Mean and standard deviation of DNA migration, expressed as percentage of tail intensity (TI%) of two independent experiments, are reported. As negative control was used the highest concentration of DMSO, as positive control was used EMS (2 mM). *p < 0.05; **p < 0.01; ***p < 0.001.

Furthermore, to identify if **L5** was able to induce subtoxic DNA alterations on human cells, its DNA damaging activity was assessed through the Alkaline Comet assay. After 1 h of exposure, **L5** produced a dose-dependent DNA migration in the Comet assay, starting from 25 µM, evidencing its genotoxic activity (Fig. 4).

## Discussion

### Chemistry

In **L1–L6** the substituents on the aromatic ring are modified in order to modulate their physicochemical features, methylation of the NH_2_ moiety in **L5** and **L6** was thought in order to modulate the lipophilicity and hydrogen-bonding capabilities. As already mentioned in the Introduction, copper salts have been long used in agriculture for their ability to inhibit the development of pathogens. On the other side, metal chelation could lead to improved bioavailability of the copper ion, facilitating its penetration into lipid membranes and thus resulting, hopefully, in better antimycotic profiles for the metal complexes. With this in mind, **L1–L4** are reacted with CuCl_2_ leading to the isolation of the copper(I) complexes **1–4**. The ^1^H-NMR spectra of **1–4**, recorded in DMSO-d_6_, support the copper(I) oxidation state for the metal ion: Cu(I), in fact, has a d^10^ electronic configuration and its diamagnetic nature led to sharp signals. In the spectrum of **1**, for example, (Fig. S1) the NH proton was shifted to lower fields, compared to the free ligand, due to the coordination to the metal; the same trend was observed for the chemical shifts of the NH_2_ and of the iminic proton. The formation of copper(I) complexes is not completely surprising: it is known that thiosemicarbazones can undergo intramolecular oxidative cyclisation in presence of bases^33^, oxidants^34^ or redox-active metals like Fe^3+^ or, as in the present case, Cu^2+^ ^35^. Copper(II) reduction is a step of a mechanism in which the cyclisation of part of the thiosemicarbazone ligand is also involved^36^. Since thiosemicarbazones possess two nucleophilic centres (N(5) and S) and a C = N double bond, two principal mechanisms are possible, leading to different products: 1,2,4-triazole-3-thione derivatives, formed by intramolecular addition of N(5) to C = N (Fig. 2A), and 1,3,4-thiadiazoline-2-amine derivatives, obtained by the addition of the sulphur atom to the iminic moiety (Fig. 2B). Other oxidative cyclisation mechanisms are reported and desulfurization processes can also occur^37^. However, in the ^1^H-NMR spectra of **1–4** no signals attributable to the cyclized products could be seen. The signals related to the cyclized ligand can be found in ^1^H-NMR spectra of a solid obtained by evaporation of the mother liquor after precipitation of the complex (as an example, see Fig. S2). No signals related to the iminic proton are present in this spectrum, as well as peaks attributable to the NH_2_ protons, indicating that part of the ligand has undergone a cyclization process according to Fig. 2A. As a consequence of the copper(II) reduction and the cyclization processes, the initial 2:1 ligand to metal stoichiometry is lost and the multimetallic complexes **1**–**4** are obtained. The formation of these structures could be possible because the thiosemicarbazone ligands possess various coordinative sites: **L1–L4** can coordinate the metal ion as monodentate, *N*,*S*-bidentate or can form bridged structures. The presence of other donor atoms on the phenyl ring could give rise to additional coordination sites. In particular, data analysis suggests 3:2 metal to ligand stoichiometry for compounds **1**, **3** and **4**, and 2:1 metal to ligand stoichiometry for complex **2**. In all the ESI-MS spectra peaks relative to multimetallic species are present, even if with very low intensity (Fig. S3–S6), while base peaks are relative to the 2:1 ligand to metal species. Comparison of the IR spectra of the copper complexes with that of the corresponding ligands made it possible to highlight a shift to slightly higher wavenumbers of the C = N stretching vibration, indicating the involvement of the iminic nitrogen in the coordination to the metal ion. It seems reasonable to conclude that the reactions of **L1–L4** with CuCl_2_ led to the isolation of multimetallic copper(I) complexes but, unfortunately, hypotheses on the structures of these complexes **1**–**4** still remain speculative due to the absence of X-ray single crystal diffraction data.

Different is the case of the ligands **L5** and **L6** (Fig. 1), where the NH_2_ hydrogens are substituted by two methyl groups. In these cases, in fact, during the reactions with CuCl_2_, reduction of copper(II) is accompanied by complete cyclisation of the ligands to obtain the Cu(I) complexes **5** and **6**. In their ^1^H-NMR spectra the aromatic region displayed sharp signals related to the hydrogens of the phenyl ring of the coordinated cyclized thiosemicarbazone. No other signals can be found around 8 or 10 ppm, related respectively to the iminic or NH protons: **L5** and **L6** are cyclized according to Fig. 2B.

### X-ray structure analysis

The crystal structure of **L5**′, the cyclized form of **L5**, is represented as an ORTEP view in Figure S7. The molecule is formed by two moieties, the benzene and the thiadiazolic rings. Both fragments are planar but not perfectly coplanar: in fact, the two planes present a slight tilt of 11.12° around the C2-C3 bond. In the aromatic 1,3,4-thiadiazolic ring, the double bonds are fairly localized between C1 = N2 and C2 = N3 (1.321 and 1.304), in contrast with 1.373 A of the N-N bond and 1.745 and 1.750 of the C-S bonds which are closer to single bond lengths. The packing is mainly determined by pairs of CH…N hydrogen bonds between two aromatic carbon atoms and the two nitrogen atoms of the thiadiazolic ring of a nearby molecule. This system of hydrogen bonds forms ribbons which propagate along the *c* axis direction. The ribbons are in turn packed through an extended network of van der Waals interactions between the methyl groups and the aromatic rings of adjacent molecules.

In the crystal structure of compound **5**′ (Fig. 3A) the copper(II) ion lies on a centre of symmetry and is surrounded by two chloride ions and two ligands, bonded through the N2 nitrogen, in a square planar coordination geometry. The ligands are almost perpendicular to the coordination plane forming an angle of 78.30°. Noteworthy are the intermolecular interactions between the terminal methyl groups and the chlorine atoms, which characterize the packing, as shown in Figure S8.

Similarly the crystal structure of **6**′ is formed by a copper(II) ion lying on a centre of symmetry in a square planar coordination geometry surrounded by two chloride ions and two ligands (Fig. 3B). Differently from **5**′, the atom bonded to copper is the N3 nitrogen. Also in this case the ligands are almost perpendicular to the coordination plane and form an angle of 87.43°. In this structure the most characterizing feature in the packing is the hydrogen bonds between the oxygens of the methoxy groups and the terminal methyl groups of an adjacent molecule (Fig. S9).

### Fungal growth and aflatoxin inhibition

Looking at the results reported in Table 1, it can be seen that among the free ligands, only **L2** (at 50 μM) and **L5** (at 100 μM) halved the biomass increase, while **L1**, **L3**, **L4** and **L6** were provided with no or scarce fungistatic activity.

**L5** and **L6** showed the best results in term of inhibition of mycotoxin biosynthesis (Table 1). Interestingly, as reported above, **L5** displayed a moderate fungistatic activity (43 and 22% at 100 and 50 μM, respectively). This is an important aspect to take into consideration: indeed the prevalent economic and sanitary issue posed by *A*. *flavus* colonization of cereal crops is essentially dependent on mycotoxin release by the mould on the contaminated substrate rather than to a plant pathogenic effect. Specifically targeting the aflatoxin biosynthetic apparatus of *A*. *flavus*, by using a compound with as low as possible generic fungistatic activity, may have a not secondary beneficial effect of avoiding possible deleterious outcomes due to unwanted modification of the microbiota composition in the environment.

Differences in activity on AF production can be analyzed as a function of the lipophilicity of the compound, as lipophilicity is generally correlated to the ability of the molecule to penetrate through the cell membrane^38, 39^. Also in this case, the low activity of **L1–L4** could be correlated to their inability to efficiently penetrate the cell membrane due to their low values of lipophilicity (Table S1); **L5** and **L6** are more lipophilic and an increased activity was observed.

Coordination to copper ions generally increases antiaflatoxigenic activity, resulting in a better activity profile (Table 1).

### Cytotoxicity and genotoxicological assessment on bacteria, plants and human cells

Fungal and aflatoxigenic inhibitors have to be used in agriculture and, obviously, they have to be safe for the operators and for the environment. Thus, a screening of the cytotoxicity of the most active compounds in term of aflatoxin inhibition (**2**, **3**, **5**, **6** and **L5**) was performed over a panel of human cell lines. Three normal cell lines were taken into consideration: colon (CRL1790), skin (Hs27), lung (HFL1), and one tumoral cell line (U937). Normal cells were chosen to represent the different routes of exposure by which this kind of chemicals can come in contact and/or enter human bodies: epidermal contact (Hs27), inhalation (HFL1) and ingestion (CRL1790). In addition, we used a tumoral cell line (U937), since it is a good cell model used worldwide to identify cytotoxicity and genotoxic activity of drugs^40^. Looking at the results (Table 2 and Figure S10) it can be seen that only **L5** has a good cytotoxicity profile, while, unfortunately, all the copper complexes showed important cytotoxicity in the micromolar range, and cannot be further evaluated.

**L5**, instead, was selected for analysis of mutagenicity and genotoxicity on bacteria, plants and human cells. The mutagenic action of a hit compound, in fact, has to be carefully evaluated for the development of new agrochemicals safe for the environment and human health. As reported in Table 3, **L5** exhibited no mutagenicity in the Ames test on *S*. *typhimurium* TA100 and TA98 strains with or without metabolic activation at all tested doses. However, even if we could not find a clear genotoxic effect of **L5** with *A. cepa* tests, the chromosomal damage that we observed at 25 µM dose suggested a potential clastogenic or aneugenic activity. Moreover, **L5** toxic activity was revealed by the inhibition of cell cycle, showed by decrease of mitotic index at the highest dose (Table 4). Since it is important to establish also the potential ability of a new hit compound to induce damage of human cellular DNA, an Alkaline Comet assay was performed on **L5**. Results reported in Fig. 4 unfortunately confirm the data collected on *A. cepa*, showing a damaging activity of **L5** on DNA that could be correlated to chromosomal aberrations induction.

## Conclusions

In the present work, the thiosemicarbazone ligands **L1–L6** have been evaluated for their antifungal and antiaflatoxigenic ability. Effectively, some of these compounds showed relevant aflatoxigenic inhibition (up to 90% reduction of AF accumulation for **L5** at 100 μM), still maintaining a moderate fungistatic activity. This last aspect is particularly important. In fact, specifically targeting the aflatoxin biosynthetic process, by using a compound with as low as possible fungistatic activity, may avoid possible deleterious outcomes due to unwanted modification of the microbiota composition in the environment. The antifungal and antiaflatoxigenic activities vary considerably along the series **L1–L6**, without apparent connection with the complexing ability of these molecule, an idea that, on the contrary, it is often invoked in the literature^41^. Perhaps, the differences in activity in **L1–L6** could be related to variations in lipophilicity, since an increase of lipophilicity seems to imply an increase in antiaflatoxigenic activity. It is claimed^12^ that metal complexation leads to species more active than the corresponding ligand in term of both fungistatic and aflatoxigenic profile. This is also the case of the copper complexes **1**–**6** here synthesised. However, in the development of crop-protective agents, it is not sufficient to obtain efficient aflatoxigenic inhibitors, but at the same time risks for humans and plants have to be taken into account^42^. Unfortunately, all the metal complexes **1**–**6** evidenced high cytotoxicity on different human, normal and tumoral, cell lines. On the contrary, **L5** joins interesting fungistatic and antiaflatoxigenic activities, with a good cytotoxic profile and it seems a promising starting point for the development of efficient crop-protective agents. However, further studies are ongoing, because deeper genotoxical assessments on human cells and plants (Comet assay, chromosome aberration test in *A*. *cepa*) highlighted possible chromosomal aberrations induction for **L5**.

We would like to conclude that: 1) the thiosemicarbazone scaffold seems a promising chemotype for the development of aflatoxin inhibitors, and 2) claims about the individuation of efficient aflatoxin inhibitors, in particular if metal complexes, for further developments as crop-protective agents, have to be combined with deep studies to assess the genotoxic potential risk for environmental and human health.

## Electronic supplementary material

Supplementary Information
