# Supplementary material for: Thiosemicarbazone scaffold for the design of antifungal and antiaflatoxigenic agents: evaluation of ligands and related copper complexes
Source: Sci Rep. 2017 Sep 11;7:11214. doi: 10.1038/s41598-017-11716-w (PMC5593876; doi:10.1038/s41598-017-11716-w)
Supplement: Supplementary file 1 — Supplementary Information [file 41598_2017_11716_MOESM1_ESM.pdf]

Thiosemicarbazone scaffold for the design of antifungal and antiaflatoxigenic agents: evaluation of ligands and related copper complexes

Dominga Rogolino,*[‡] Anna Gatti,[‡] Mauro Carcelli,[‡] Giorgio Pelosi,[‡] Franco Bisceglie,[‡] Francesco Maria Restivo,[‡] Francesca Degola,[‡] Annamaria Buschini,[‡] Serena Montalbano,[‡] Donatella Feretti,[±] and Claudia Zani[±]

[‡]*Department of Chemistry, Life Sciences and Environmental Sustainability and CIRCMSB (Consorzio Interuniversitario di Ricerca in Chimica dei Metalli nei Sistemi Biologici), Università di Parma, Parco Area delle Scienze, 43124 Parma, Italy.* [±]*Department of Medical and Surgical Specialities, Radiological Sciences and Public Health, University of Brescia, Viale Europa 11, 25123 Brescia, Italy*

Figure S1. Comparison between the ^{1}H-NMR spectrum of the ligand **L1** (violet) and of the corresponding complex **1** (red).

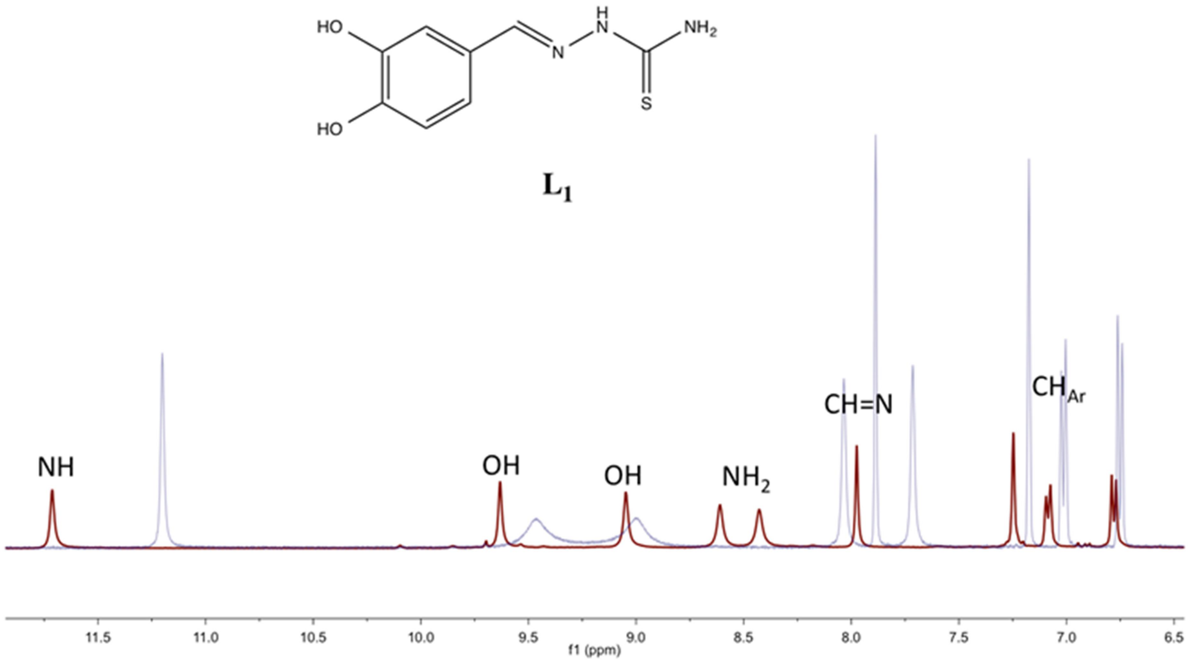

Figure S2. ^{1}H-NMR spectrum of residues of the mother liqueur of complex **3.**

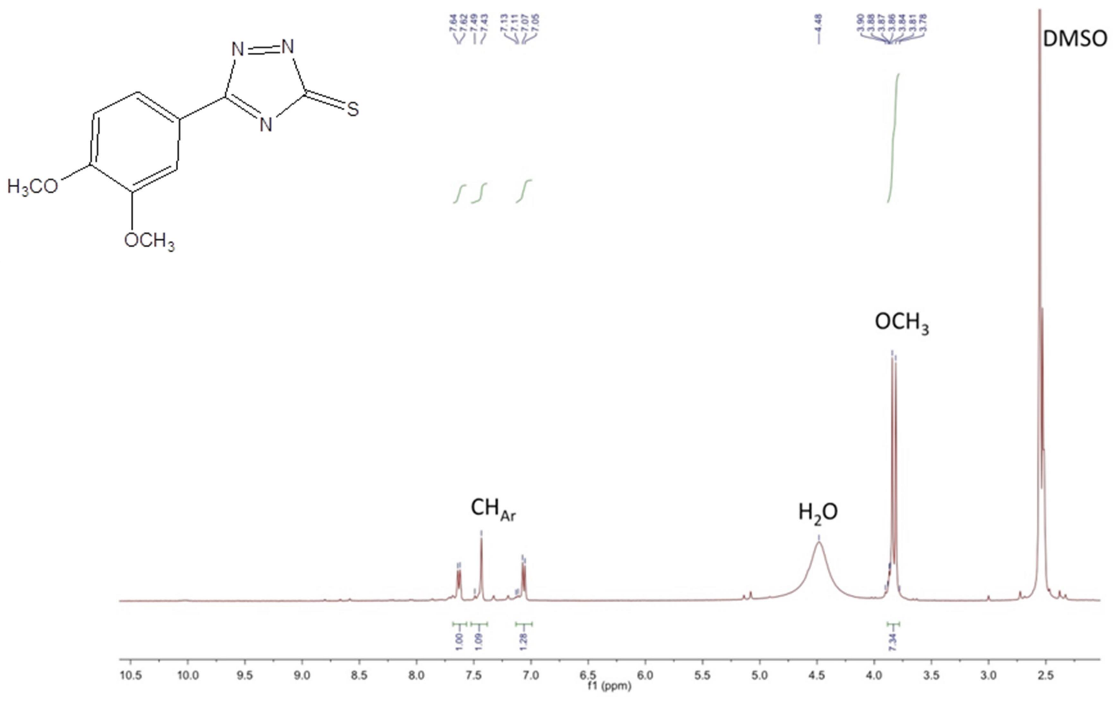

Figure S3. ESI(+) spectra of a methanol solution of **1**. The range m/z 500-1000 was magnified (x4) to evidence the multinuclear peaks of lower intensity.

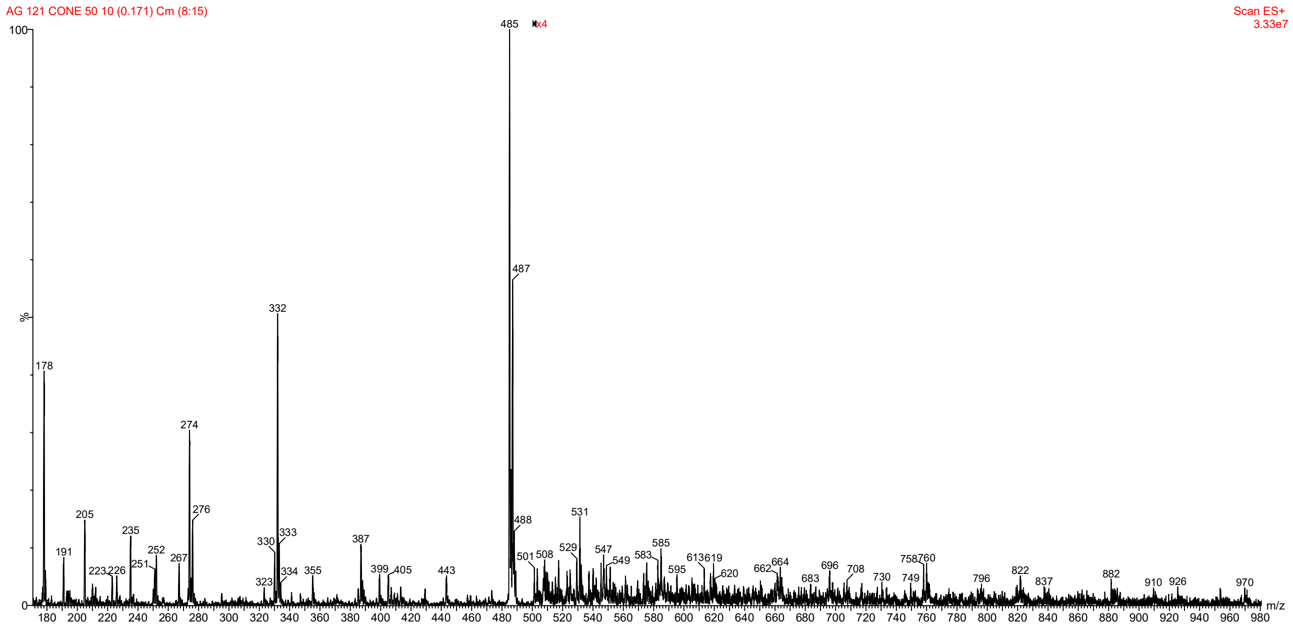

Figure S4. ESI(+) spectra of a methanol solution of **2**.

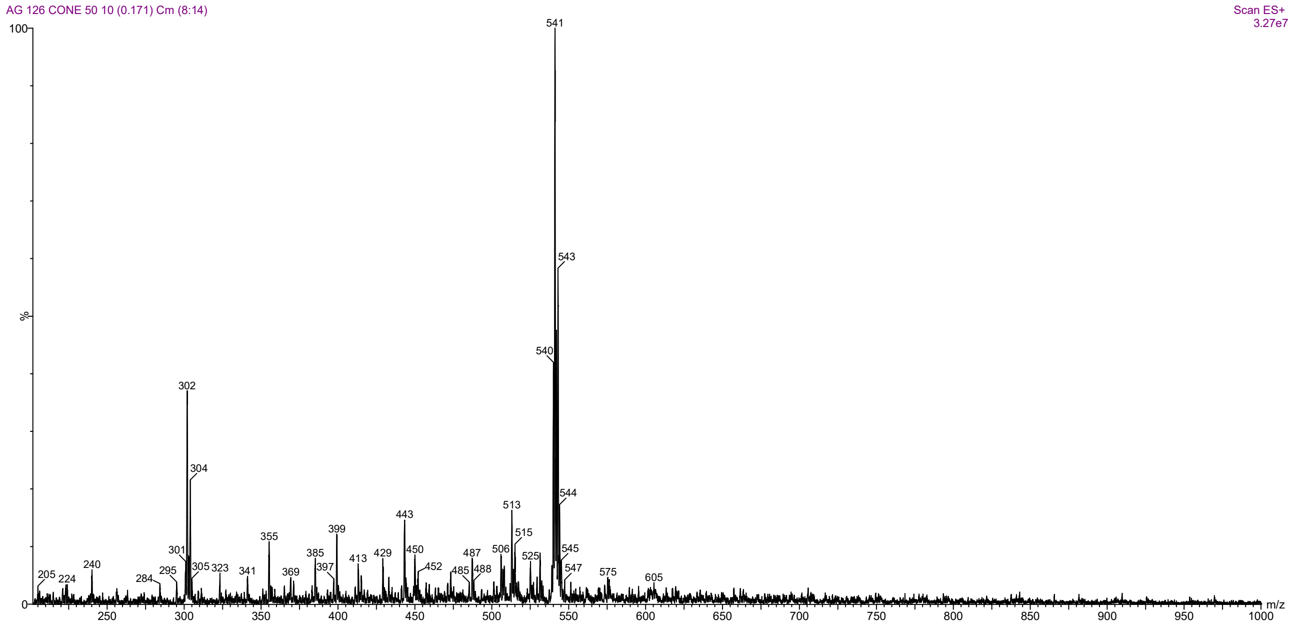

Figure S5. ESI(+) spectra of a methanol solution of **3**.

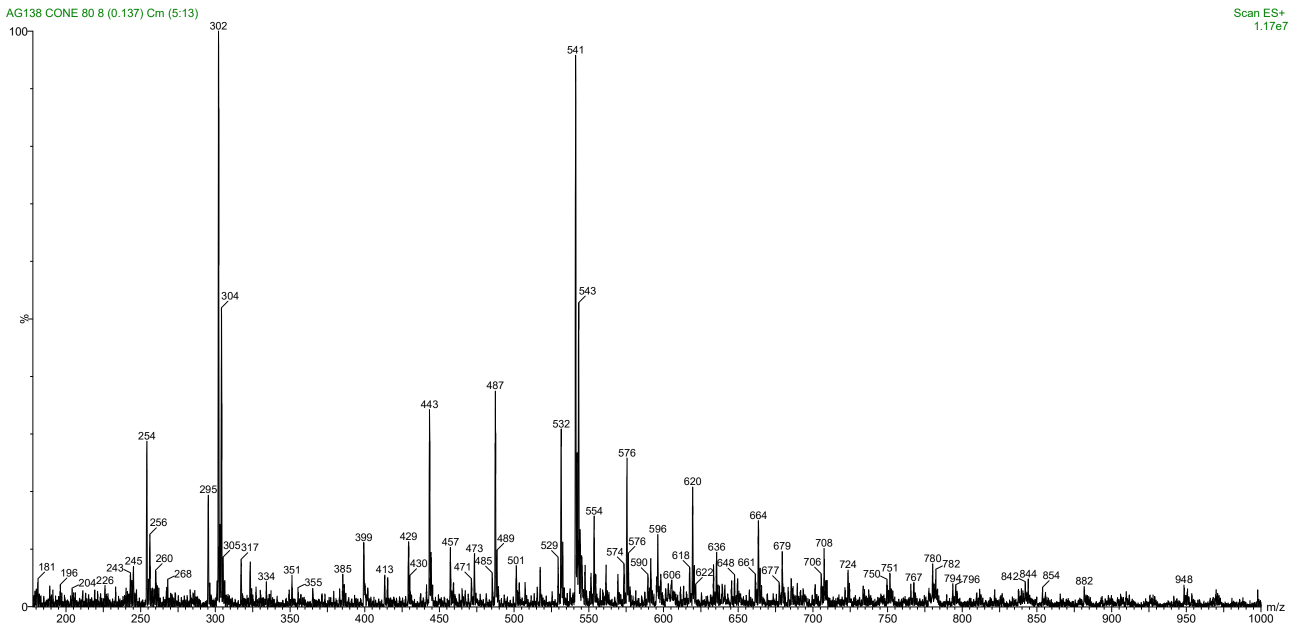

Figure S6. ESI(+) spectra of a methanol solution of **4**. The range m/z 500-1000 was magnified (x4) to evidence the multinuclear peaks of lower intensity.

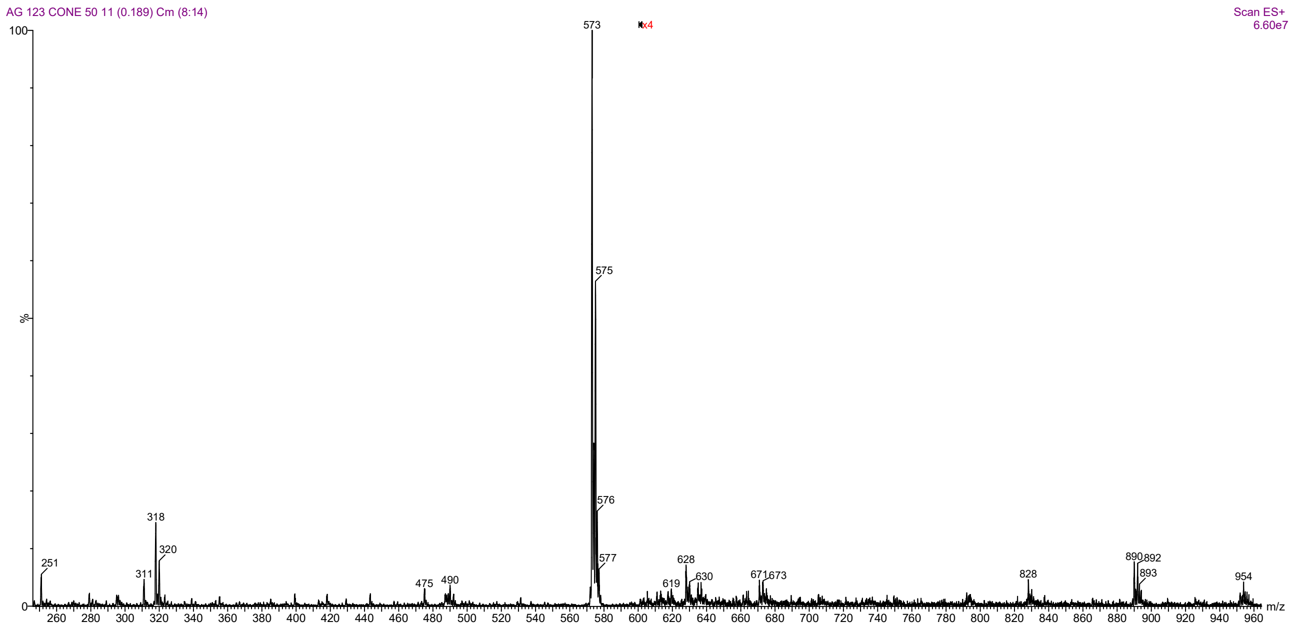

Figure S7. ORTEP representation of **L5’** with ellipsoids at 50% probability

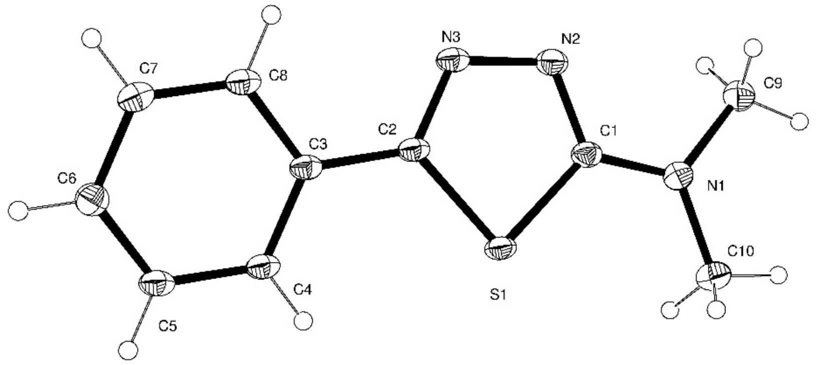

Figure S8. The packing of compound **5’** is characterized by 1D-ribbons formed through hydrogen bonds connecting terminal methyl groups with coordinated chlorine atoms of adjacent molecule running along the x direction.

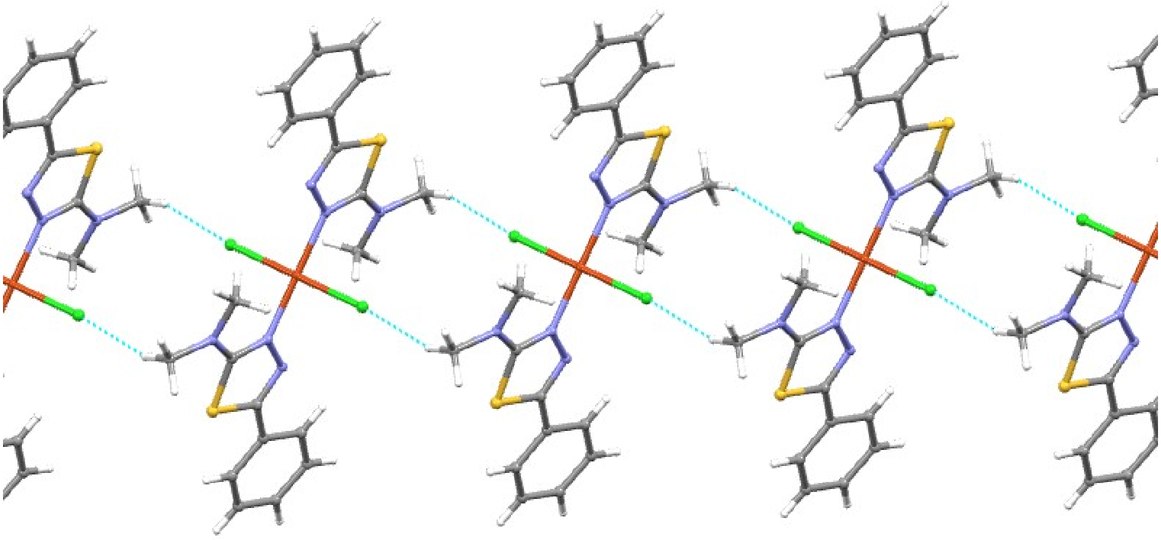

Figure S9. The packing of compound **6'** is determined by H-bonds interactions between the two terminal methyl groups and the methoxy oxygens that protrude from the aromatic ring. Also in this case, the molecules form a 1D ribbon which runs along the xz direction.

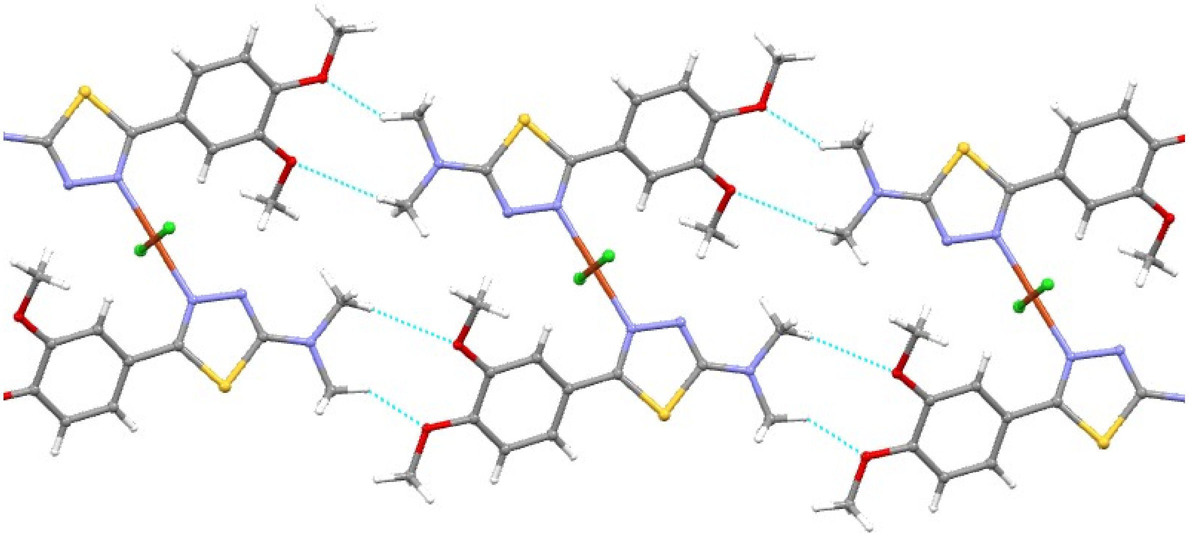

Figure S10. Dose response curve for **L5** and the copper complex **5** in Crl1791, Hs27, HFL1 and U937 cell lines. GI%: percent of growth inhibition. Negative control (dose 0): DMSO (100µM)

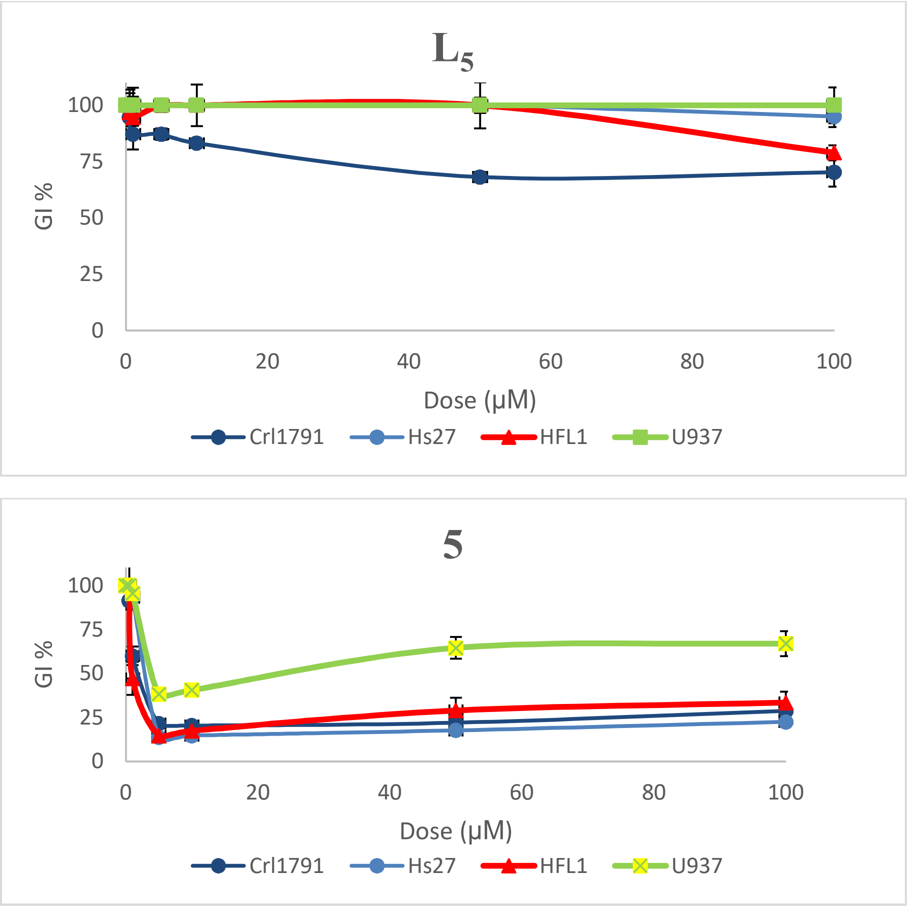

Table S1. Calculated LogP values for **L1-L6**.

Compound	LogP
**L1**	0.91
**L2**	1.14
**L3**	1.53
**L4**	1.24
**L5**	2.50
**L6**	2.15
